# Geographical and temporal trends of HIV-1 subtypes and drug resistance in China: a nationwide study over two decades (2003–2024)

**DOI:** 10.1099/jgv.0.002217

**Published:** 2026-02-02

**Authors:** Bin Xie, Zhenggang Wang, Qixiang Song, Miao Cui, Yelin Deng, Fang Zheng, Ewelina Krol, Ricardo Khouri, Erik De Clercq, Juan Mo, Guangdi Li

**Affiliations:** 1 Hunan Provincial Key Laboratory of Clinical Epidemiology, Xiangya School of Public Health, Central South University, Changsha, PR China; 2 Center for Public Health and Epidemic Preparedness & Response, Peking University, Beijing, PR China; 3 Department of Civil and Environmental Engineering, Soochow University, Suzhou, PR China; 4 Department of Infectious Diseases, The First Hospital of Changsha, The Affiliated Changsha Hospital of Xiangya School of Medicine, Central South University, Changsha, PR China; 5 Department of Recombinant Vaccines, Intercollegiate Faculty of Biotechnology, University of Gdansk and Medical University of Gdansk, Gdansk, Poland; 6 Faculdade de Medicina, Universidade Federal da Bahia, Salvador, Bahia, Brazil; 7 Laboratório de Medicina e Saúde Pública de Precisão, Instituto Gonçalo Muniz, Fundação Oswaldo Cruz, Salvador, Bahia, Brazil; 8 KU Leuven, Department of Microbiology, Immunology and Transplantation, Rega Institute, Leuven 3000, Belgium

**Keywords:** human immunodeficiency virus 1 (HIV-1), subtype distribution, transmitted drug resistance

## Abstract

**Objective.** This study aims to characterize the 20-year trends in human immunodeficiency virus 1 (HIV-1) subtypes and circulating recombinant forms (CRFs) in China, as well as patterns of transmitted drug resistance (TDR) to antiretroviral therapies commonly used in clinical practice.

**Methods.** We analysed HIV-1 sequences from 81,563 individuals living in China between 2003 and 2024. Subtypes and CRFs were classified using COMET V2.4. Among these, *pol* gene sequences from 41,486 treatment-naïve individuals were used to assess TDR via the Stanford HIVdb genotypic resistance interpretation program.

**Results.** Over the past two decades, CRF01_AE (43.9%) was the most prevalent HIV-1 strain in China, followed by CRF07_BC (19.0%), subtype B (12.3%), subtype C (8.4%) and CRF08_B (4.9%). However, subtype and CRF distributions varied considerably across provinces. CRF01_AE predominated in provinces such as Liaoning (92.4%), Guangxi (58.7%), Beijing (47.7%) and Hainan (44.8%), while CRF07_BC was dominant in Sichuan (63.6%), Chongqing (53.2%) and Xinjiang (82.7%). TDR analysis revealed elevated resistance to non-nucleoside reverse transcriptase inhibitors in certain provinces, including Yunnan (12.4%), Xinjiang (8.2%), Anhui (7.6%) and Henan (6.7%). In contrast, resistance to nucleoside reverse transcriptase inhibitors and integrase inhibitors remained low (<1%) across all regions. Notably, the TDR rate exceeded 5% for several regimens freely provided in China, including AZT+3TC+NVP (6.8%), AZT+3TC+RPV (8.0%), AZT+3TC+EFV (6.4%), TDF+3TC+NVP (6.0%), TDF+3TC+RPV (7.2%) and TDF+3TC+EFV (5.7%).

**Conclusion.** Continued surveillance of HIV-1 genotypes and CRFs is critical, particularly in regions where routine genotypic testing is not implemented. Personalized antiretroviral regimens are urgently needed in regions with high levels of TDR.

## Data Summary

All data that support the findings of this study are openly available in the Los Alamos National Laboratory HIV Sequence Database (https://www.hiv.lanl.gov/content/index), the NCBI nucleotide database (https://www.ncbi.nlm.nih.gov/nucleotide/) and the Stanford HIVdb Program (https://hivdb.stanford.edu/hivdb/by-patterns/).

## Introduction

By the end of 2024, nearly 40.8 million people were living with human immunodeficiency virus (HIV) worldwide, according to the World Health Organization. Approximately 31.6 million were receiving antiretroviral therapy. In China, ~1.329 million people will be living with HIV by the middle of 2024, with more than 90% receiving antiretroviral therapy [1]. Since the adoption of the ‘treat-all’ policy in June 2016 which provides antiretroviral therapies to all patients regardless of CD4^+^ T-cell count [2], all-cause mortality among people with HIV in China has decreased from 5.4% in 2013 to 2.7% in 2022 [3]. Despite the effectiveness of antiretroviral therapies, drug resistance remains a major challenge to the control of HIV/acquired immune deficiency syndrome globally [4].

Many antiretroviral therapies have been approved for the treatment of human immunodeficiency virus 1 (HIV-1) infection [5,6]. In China, the Fifth Edition of the National Guidelines for Free Antiretroviral Therapy [7] serves as the national standard. According to this guideline, freely available regimens typically consist of two nucleos(t)ide reverse transcriptase inhibitors (NRTIs) in combination with a non-nucleoside reverse transcriptase inhibitor (NNRTI), a protease inhibitor (PI), or an integrase strand transfer inhibitor (INSTI). Acquired or transmitted drug resistance (TDR) has been widely reported among people living with HIV-1 in China. A meta-analysis of drug resistance data from 2001 to 2017 reported a high prevalence of acquired resistance to NRTIs (31.4%), NNRTIs (39.5%) and PIs (1%) [8]. Regional variations were also observed; for instance, acquired NNRTI resistance was higher in central China than in northern or southern China [8]. Among 2,568 treatment-naïve individuals with HIV-1 infection, the TDR rate to NNRTIs, NRTIs and PIs was 6.3%, 1.2% and 0.2%, respectively [9]. Moreover, TDR exhibited regional discrepancy and some provinces (e.g. Sichuan) had notably high resistance [9]. Beyond drug selective pressure, differences in HIV-1 subtypes and circulating recombinant forms (CRFs) also contribute to variability in drug resistance [10,11]. Therefore, timely surveillance is essential to guide treatment optimization and improve outcomes [12].

Our previous study reported HIV-1 drug resistance in South-Central China [13] and summarized the clinical efficacy of antiretroviral therapies [5,6, 14, 15]. Although previous studies explored HIV-1 subtype distribution and drug resistance in selected regions in China, no nationwide study has examined the 20-year trends in HIV-1 subtypes/CRFs and drug resistance from a nationwide perspective. Furthermore, previous studies mainly focused on resistance to individual antiviral inhibitors, rather than resistance patterns associated with the commonly used regimens of antiretroviral agents freely provided in China. Using a large-scale dataset of HIV-1 sequences from 2003 to 2024, this study provides a comprehensive analysis of subtype/CRF distribution and TDR to the most widely used, freely provided regimens in China. Our findings will shed light on the trend of subtype distributions and HIV-1 drug resistance in China, contributing to HIV-1 surveillance and optimized treatment strategies.

## Methods

### Sequence collection

To compile a large-scale sequence dataset, we extracted HIV-1 sequences sampled in China between 1 January 2003 and 31 December 2024 from the HIV Databases at Los Alamos National Laboratory (https://www.hiv.lanl.gov/) and the NCBI nucleotide database (https://www.ncbi.nlm.nih.gov/nucleotide/). HIV-1 sequences were extracted and screened based on the following criteria: one sequence per patient and the presence of the *pol* gene. We collected information on the patient ID, sampling year, the sampling location, treatment-naive status and subtypes from the Los Alamos National Laboratory HIV Sequence Database (https://www.hiv.lanl.gov/content/index). In order to supplement the dataset, we retrieved the HIV sequences of each province in China from the NCBI (https://www.ncbi.nlm.nih.gov/). The final dataset included *pol* sequences from 81,563 people living with HIV-1 infection, of which 41,486 were from treatment-naïve individuals. Due to the missing data, subsets with complete metadata were used for year-specific (*N*=62,554) and province-specific (*N*=55,708) analyses.

### HIV-1 genotyping

Multiple sequence alignment was conducted using the MAFFT V7.5 (https://mafft.cbrc.jp/) with HXB2 strain as the reference sequence. HIV-1 genotypes were determined using the COMET V2.4 toolkit (https://comet.lih.lu) and the NCBI genotyping tool (https://www.ncbi.nlm.nih.gov/labs/virus). Discordance results were resolved using the REGA HIV-1 and 2 automated subtyping tool V2.0. In our analysis of HIV-1 subtype/CRF proportions, a limited number of sequences (*N*<50) were available in certain provinces, including Inner Mongolia, Tibet, Gansu, Ningxia and Jiangxi. Due to the incomplete sharing of HIV-1 sequences in public databases, we conducted a comprehensive literature search of peer-reviewed publications in both English and Chinese. Sources included PubMed, Google Scholar, Baidu Scholar, Wanfang Data and the China National Knowledge Infrastructure (CNKI). The literature findings from sequence analyses are summarized in Table S1 (available in the online Supplementary Material).

### Analysis of TDR

TDR was analysed using HIV-1 pol sequences from 41,486 treatment-naïve individuals. Most sequences (92.0%) included only the protease and reverse transcriptase regions and lacked the integrase region. Therefore, NRTI, NNRTI and PI resistance was assessed in all 41,486 sequences, whereas INSTI resistance was analysed in the subset containing the integrase region (*N*=3,326). For each sequence, its resistance score was measured for five NRTIs (TDF, 3TC, AZT, ABC and FTC), three NNRTIs (EFV, RPV and NVP), two INSTIs (DTG and RAL) and one PI (LPV) using the mutation analysis from the HIVdb program at the HIV Drug Resistance Database (https://hivdb.stanford.edu/hivdb/by-patterns/). Each antiretroviral drug above was freely available in China [7]. Based on the HIVdb program, any sequence with a resistance score ≥15 to a specific drug is classified as a drug-resistant sequence to this drug. Resistance to three-drug regimens was defined as resistance to ≥1 drug component. By using the HIVdb program, the resistance score was calculated for each sequence and the cutoff of ≥15 was used to determine drug resistance for a given antiretroviral therapy. The NNRTI resistance rate was defined by the proportion of sequences with transmitted resistance to any NNRTI (resistance score ≥15). The resistance rates of NRTIs, PIs and INSTIs were similarly defined.

### Statistical analysis

Descriptive analyses were conducted to report the geographic and temporal distribution of HIV-1 subtypes. The ggplot2 package in R was employed to visualize the dynamics in HIV-1 subtype distribution across different sampling years. Statistical analyses were performed using R 4.3.3.

## Results

### Temporal dynamics of HIV-1 subtypes and CRFs in China

**Fig. 1.**
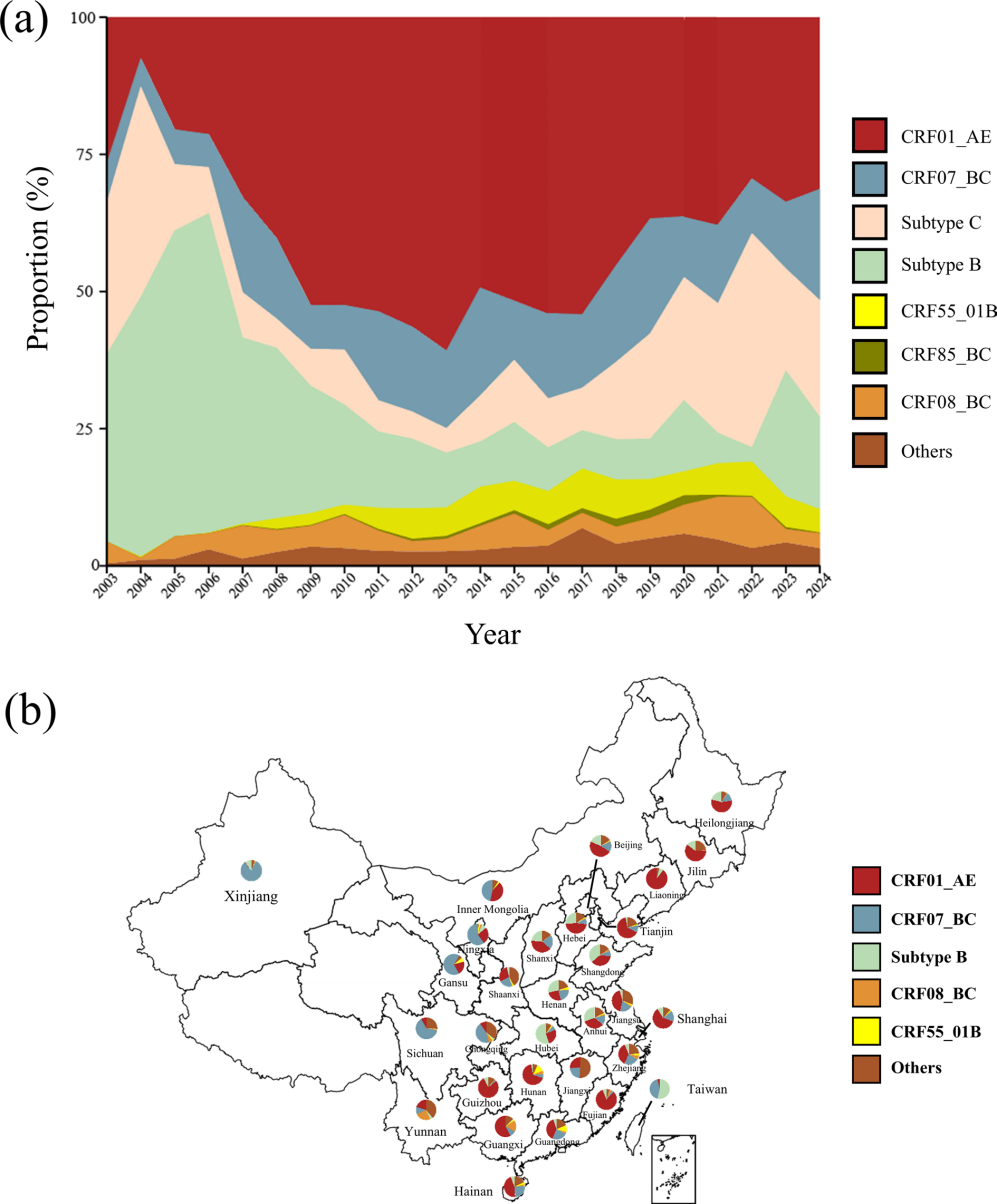
Temporal trends in HIV-1 subtypes and CRFs from 2003 to 2024. (**a**) Proportions of major HIV-1 subtypes and CRFs based on HIV-1 sequences from 81,563 individuals living in China. The x-axis covers the years from 2003 to 2024. The y-axis shows the cumulative proportions of circulating HIV-1 subtypes and CRFs using a stacked area chart. The ‘Others’ category includes minor subtypes and CRFs such as CRF101_01B, CRF100_01C, CRF 33_01B and CRF 57_BC. (**b**) Distribution of HIV-1 subtypes and CRFs across provincial-level regions, based on 62,554 sequences with the sampling locations. To reveal geographic heterogeneity, a pie chart was generated for each provincial-level region. Several regions, including Tibet, Qinghai, Gansu, Jiangxi, Ningxia and Inner Mongolia, were excluded due to the absence of sequence data.

Based on a large-scale dataset of HIV-1 sequences from 81,563 individuals living in China, we analysed HIV-1 subtype/CRF distributions from 2003 to 2024 (Fig. 1a). CRF01_AE (43.9%) was the most prevalent HIV-1 strain in China, followed by CRF07_BC (19.0%), subtype B (12.3%), subtype C (8.4%) and CRF08_B (4.9%) and others. In 2003, the proportions of CRF01_AE (26.6%), subtype B (34.1%) and subtype C (22.2%) are relatively similar. The subtype B was the dominant strain from 2003 to 2007, but its proportion steadily declined from 47.3% in 2004 to 16.2% in 2024 (Fig. 1a). The proportion of CRF01_AE increased from 26.6% in 2003 to 60.8% in 2013 and then declined in recent years (Fig. 1a). Between 2020 and 2024, CRF01_AE (33.4%), subtype C (17.5%) and CRF07_BC (19.6%) were the most prevalent. Other minor variants such as CRF09_cpx only accounted for a smaller proportion (<1% each), but their overall growth showed gradual increases after 2009 (Fig. 1a).

### Geographical distribution of HIV-1 subtypes and CRFs in China

After excluding the entries with missing geographic information from the dataset of 81,563 sequences, we conducted province-level analyses (*N*=62,554 sequences) to reveal geographic heterogeneity (Table 1). Distribution of HIV-1 subtypes and CRFs exhibited a diverse pattern in different provinces in China (Fig. 1b). Among 30 provincial-level regions with available sequences, only 6 had a dominant subtype/CRF exceeding 60%, while 17 had at least 2 circulating strains exceeding 20% each (Fig. 1b). For instance, CRF01_AE (25.4%), CRF07_BC (21.2%) and subtype B (28.6%) co-circulated in the province Henan.

**Table 1.** Summary of HIV-1 subtype/CRF sequences sampled in China

Region	Sequence no.	HIV-1 subtype and CRF (%)					
		CRF01_AE	CRF07_BC	B	CRF08_BC	CRF55_01B	Others
Beijing	8,230	3,928 (47.7)	1,294 (15.7)	1,511 (18.4)	71 (0.9)	92 (1.1)	1,334(16.2)
Hebei	1,337	631 (47.2)	117 (8.8)	349 (26.1)	12 (0.9)	19 (1.4)	209 (15.6)
Shanxi	388	155 (39.9)	81 (20.9)	92 (23.7)	3 (0.8)	0 (0.0)	57 (14.7)
Tianjin	311	203 (65.3)	33 (10.6)	9 (2.9)	1 (0.3)	5 (1.6)	60 (19.3)
Shandong	105	39 (37.1)	9 (8.6)	38 (36.2)	3 (2.9)	0 (0.0)	16 (15.2)
Jiangsu	1,752	742 (42.4)	324 (18.5)	76 (4.3)	32 (1.8)	42 (2.4)	536 (30.6)
Anhui	1,128	379 (33.6)	165 (14.6)	344 (30.5)	22 (2.0)	19 (1.7)	199 (17.6)
Zhejiang	1,831	677 (37.0)	422 (23.0)	116 (6.3)	116 (6.3)	57 (3.1)	443 (24.2)
Fujian	166	136 (81.9)	5 (3.0)	10 (6.0)	6 (3.6)	0 (0.0)	9 (5.4)
Shanghai	1,409	854 (60.6)	233 (16.5)	128 (9.1)	5 (0.4)	23 (1.6)	166 (11.8)
Hubei	820	217 (26.5)	55 (6.7)	452 (55.1)	4 (0.5)	16 (2.0)	76 (9.3)
Hunan	69	46 (66.7)	4 (5.8)	2 (2.9)	4 (5.8)	8 (11.6)	5 (7.2)
Henan	4,220	1,071 (25.4)	896 (21.2)	1,208 (28.6)	51 (1.2)	169 (4.0)	825 (19.5)
Jiangxi	39	10 (25.6)	9 (23.1)	0 (0.0)	0 (0.0)	0 (0.0)	20 (51.3)
Guangdong	19,484	7,536 (38.7)	4,751 (24.4)	990 (5.1)	606 (3.1)	2,064 (10.6)	3,637 (18.7)
Guangxi	4,242	2,491 (58.7)	508 (12.0)	44 (1.0)	704 (16.6)	106 (2.5)	389 (9.2)
Hainan	1,273	571 (44.8)	321 (25.2)	76 (6.0)	31 (2.4)	48 (3.8)	226 (17.8)
Ningxia	27	2 (7.4)	2 (7.4)	2 (7.4)	20 (74.1)	0 (0.0)	1 (3.7)
Xinjiang	668	5 (0.8)	553 (82.7)	67 (10.0)	1 (0.2)	4 (0.6)	38 (5.7)
Shaanxi	150	43 (28.7)	33 (22.0)	6 (4.0)	1 (0.7)	4 (2.7)	63 (42.0)
Gansu	3	0 (0.0)	1 (33.3)	1 (33.3)	0 (0.0)	0 (0.0)	1 (33.3)
Sichuan	1,404	114 (8.1)	893 (63.6)	15 (1.1)	18 (1.3)	3 (0.2)	361 (25.7)
Chongqing	218	21 (9.6)	116 (53.2)	3 (1.4)	10 (4.6)	5 (2.3)	63 (28.9)
Yunnan	3,431	695 (20.3)	398 (11.6)	132 (3.8)	848 (24.7)	19 (0.6)	1,339 (39.0)
Guizhou	95	75 (78.9)	2 (2.1)	7 (7.4)	1 (1.1)	0 (0.0)	10 (10.5)
Inner Mongolia	2	0 (0.0)	1 (50.0)	0 (0.0)	0 (0.0)	0 (0.0)	1 (50.0)
Liaoning	1,994	1,844 (92.4)	26 (0.8)	109 (5.5)	4 (0.2)	1 (0.1)	20 (1)
Jilin	406	247 (60.8)	2 (0.5)	59 (14.5)	1 (0.3)	0 (0.0)	97 (23.9)
Heilongjiang	506	284 (56.1)	66 (13.0)	107 (21.1)	4 (0.8)	1 (0.2)	44 (8.7)
Total	55,708*	23,016	11,320	5,953	2,579	2,705	10,245

*A total of 55,708 sequences from people living with HIV-1 infection (2003 to 2024) in China included geographical information.

**Fig. 2.**
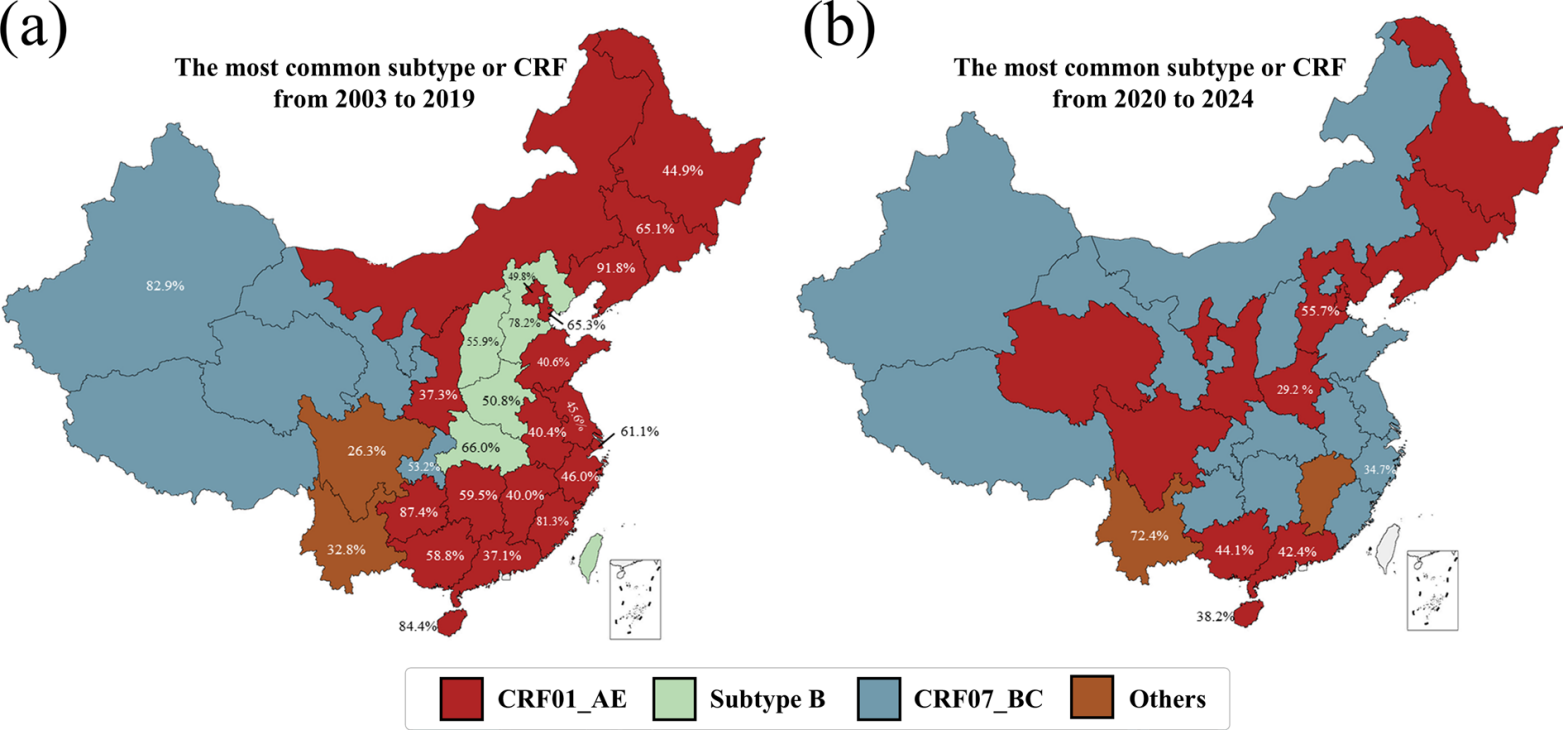
Dominant HIV-1 subtypes and CRFs in provincial-level regions of China. Dominant strains in each region are shown using HIV-1 sequences collected from 2003 to 2019 (**a**) and from 2020 to 2024 (**b**). The proportions of the dominant strains are highlighted. For regions with missing data or fewer than 30 available sequences, published data were incorporated to ensure the coverage of all provinces (Table S1).

Over the past two decades, we observed dynamic changes of the dominant HIV-1 subtypes and CRFs in different regions. From 2003 to 2019 (Fig. 2a), CRF01_AE dominated in many regions such as Liaoning province (92.5%), Tianjin (65.3%), Shanghai (61.1%), Guangxi (58.8%), Guizhou (87.4%) and Hainan (76.1%). CRF07_BC dominated in Xinjiang (82.9%) and Chongqing (53.2%). Subtype B was the dominant subtype in Hebei (78.2%), Hubei (66.0%), Henan (50.8%) and Shanxi (55.9%). Subtype C was the dominant strain in Yunnan (32.8%).

The patterns of HIV-1 subtype/CRF distributions shifted during the period of 2020 to 2024 (Fig. 2b). Changes of dominant subtypes and CRFs from the early period (2003 to 2019) to the recent period (2020 to 2024) were observed in several provinces such as Henan and Hubei. Subtype B was the dominant strain in Hebei and Henan before 2020, but CRF07_BC became the dominant strain in Hebei (55.7%) and CRF01_AE dominated in Henan (29.2%) from 2020 to 2024 (Fig. 2b).

### TDR in treatment-naive people living with HIV-1

**Fig. 3.**
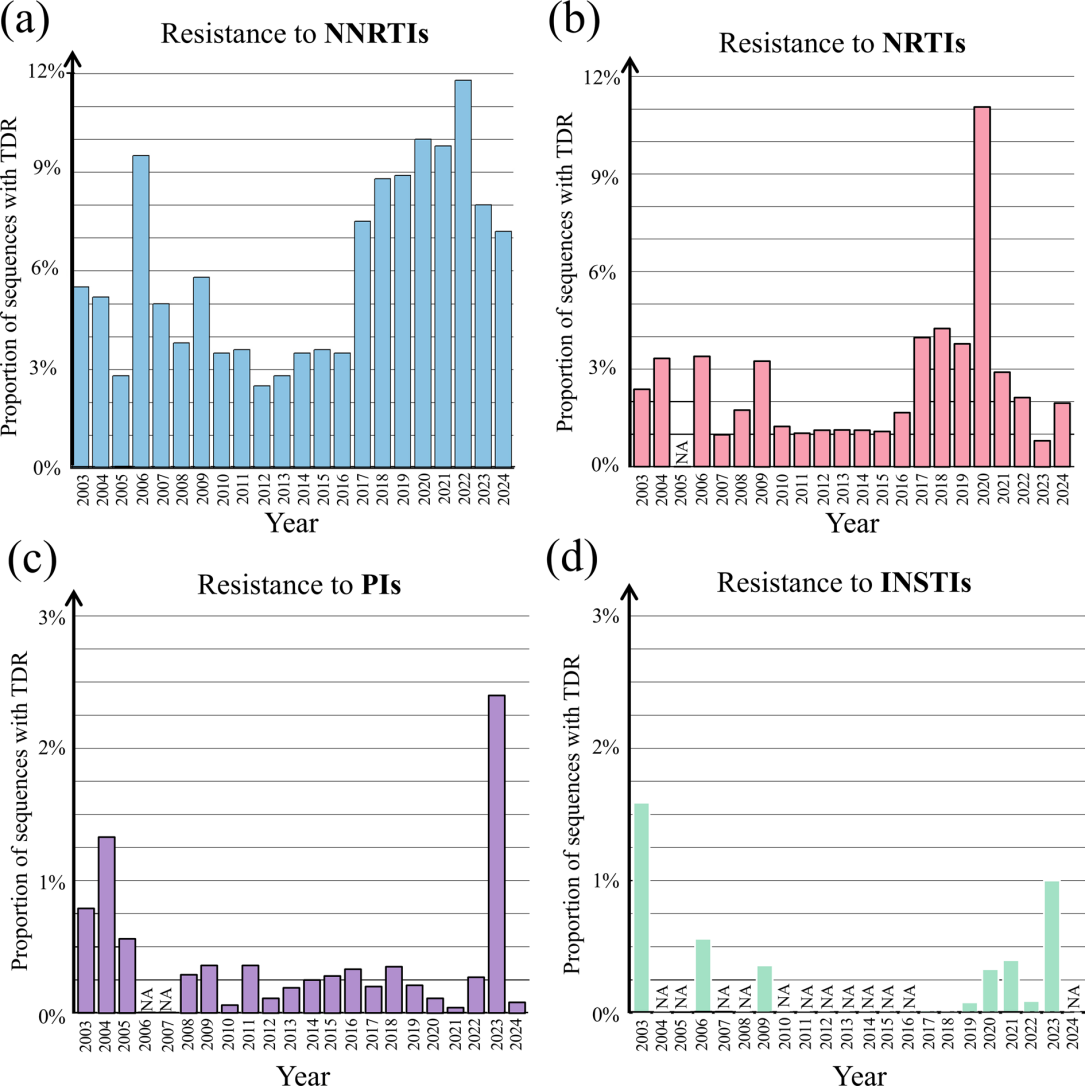
TDR to NNRTIs (**a**), NRTIs (**b**), PIs (**c**) and INSTIs (**d**). This figure shows the annual prevalence of TDR to four classes of antiretroviral drugs: NRTIs (TDF, 3TC, AZT, ABC and FTC), NNRTIs (EFV, RPV and NVP), INSTIs (DTG and RAL) and PIs (LPV). Coloured bars represent the annual proportions of HIV-1 *pol* sequences (Table S2) associated with resistance to NNRTIs (blue), NRTIs (pink), PIs (purple) and INSTIs (green). Because fewer than ten sequences were available in 2023 in our dataset (Table S2), resistance data from the publication [66] were used to supplement that year. na indicates that the data are not available.

Given a large subset of HIV-1 sequences obtained from 41,486 treatment-naive people, we analysed the transmitted resistance to five NRTIs (TDF, 3TC, AZT, ABC and FTC), three NNRTIs (EFV, RPV and NVP), two INSTIs (DTGa and RAL) and one PI (LPV) which are freely provided in China. As shown in Fig. 3(a), the transmitted resistance to NNRTIs was the highest compared with the transmitted resistance to NRTIs, INSTIs or PIs. The average resistance rates of NNRTIs, NRTIs, INSTIs and PIs were ~5.91%, 2.4%, 0.2% and 0.4%, respectively. The NNRTI resistance rate increased steadily from 2010 (3.5%) to 2024 (7.1%). In comparison, the average resistance rates of NRTI only showed a small increase from 2010 (1.0%) to 2024 (2.0%). The average INSTI resistance rate remained at a low level from 2019 (0.08%) to 2023 (1.0%).

We observed diverse patterns of transmitted resistance rates in different provinces. The TDR was mapped across provincial-level regions in China (Table 2), except for Qinghai, Guizhou and Tibet where sequences were unavailable. For those provinces with the sample size of >100 sequences, the highest NNRTI resistance rate was found in Yunnan (12.4%), followed by Xinjiang (8.2%), Anhui (7.6%), Liaoning (4.9%) and others. The highest NRTI resistance rate was observed in Liaoning (2.3%), followed by Henan (2.1%), Anhui (2%) and Tianjin (2%). In contrast, the INSTI resistance rates in all provincial-level regions were smaller than 1.5%.

**Table 2.** Summary of HIV-1 sequences with transmitted resistance to four drug classes*

Regions	Total no.	NNRTIs	NRTIs	PIs	INSTIs
		N (%)	N (%)	N (%)	N (%)
Beijing	7196	221(3.1)	56(0.8)	5(0.1)	0(0.0)
Hebei	995	26(2.6)	3(0.3)	3(0.3)	0(0.0)
Shanxi	369	14(3.8)	4(1.1)	0(0.0)	0(0.0)
Tianjin	305	13(4.3)	6(2.0)	0(0.0)	0(0.0)
Shandong	53	3(5.7)	1(1.9)	0(0.0)	0(0.0)
Jiangsu	1316	65(4.9)	11(0.8)	1(0.1)	0(0.0)
Anhui	394	30(7.6)	8(2.0)	0(0.0)	0(0.0)
Zhejiang	1582	76(4.8)	29(1.8)	6(0.4)	0(0.0)
Fujian	13	3(23.1)	1(7.7)	0(0.0)	1(7.7)
Shanghai	1318	22(1.7)	7(0.5)	1(0.1)	0(0.0)
Hubei	159	9(5.7)	3(1.9)	2(1.3)	0(0.0)
Henan	3031	203(6.7)	64(2.1)	6(0.2)	6(0.2)
Jiangxi	6	0(0.0)	0(0.0)	0(0.0)	0(0.0)
Guangdong	12068	390(3.2)	133(1.1)	28(0.2)	7(0.05)
Guangxi	3163	110(3.5)	21(0.7)	12(0.4)	0(0.0)
Hainan	73	2(2.7)	0(0.0)	0(0.0)	0(0.0)
Xinjiang	147	12(8.2)	0(0.0)	1(0.7)	0(0.0)
Shaanxi	395	15(3.8)	4(1.0)	0(0.0)	0(0.0)
Sichuan	858	28(3.3)	12(1.4)	0(0.0)	3(0.0)
Chongqing	33	0(0.0)	0(0.0)	0(0.0)	0(0.0)
Yunnan	2332	288(12.4)	23(1.0)	6(0.3)	0(0.0)
Liaoning	1363	67(4.9)	32(2.3)	0(0.0)	0(0.0)
Jilin	23	1(4.3)	0(0.0)	0(0.0)	0(0.0)
Heilongjiang	369	9(2.4)	4(1.1)	1(0.3)	0(0.0)
Ningxia	3	0(0.0)	0(0.0)	0(0.0)	0(0.0)
Hunan	2	0(0.0)	0(0.0)	0(0.0)	0(0.0)
Inner Mongolia	1	0(0.0)	0(0.0)	0(0.0)	0(0.0)
Gansu	1	0(0.0)	0(0.0)	0(0.0)	0(0.0)
Total	37,568**	1607	422	72	17

* Transmitted resistance to a given drug class was defined as a resistance score ≥15 for any compound from that drug class, including NRTIs (TDF, 3TC, AZT, ABC, FTC), NNRTIs (EFV, RPV, NVP), two INSTIs (DTG, RAL) and PI (LPV/r). **In our cohort of *pol* gene sequences, only 37,568 sequences from treatment-naïve individuals contained geographical information.

### Transmitted resistance to antiretroviral regimens recommended in China

Twelve freely available regimens have been recommended in China for the treatment of HIV-1 infections [7]. By calculating the transmitted resistance score, we estimated the resistance to 12 recommended regimens in treatment-naïve people living with HIV-1 infection. Using 41,486 sequences encoding the protease and reverse transcriptase, the average rates of transmitted resistance were measured for AZT+3TC+NVP (6.8%), AZT+3TC+RPV (8.0%), AZT+3TC+EFV (6.4%), TDF+3TC+NVP (6.0%), TDF+3TC+RPV (7.2%), TDF+3TC+EFV (5.7%), TDF+3TC+LPV/r (1.9%), AZT+3TC+LPV/r (2.8%), AZT+3TC+DTG (0.22%), AZT+3TC+RAL (0.33%), TDF+3TC+DTG (0.18%) and TDF+3TC+RAL (0.33%).

**Fig. 4.**
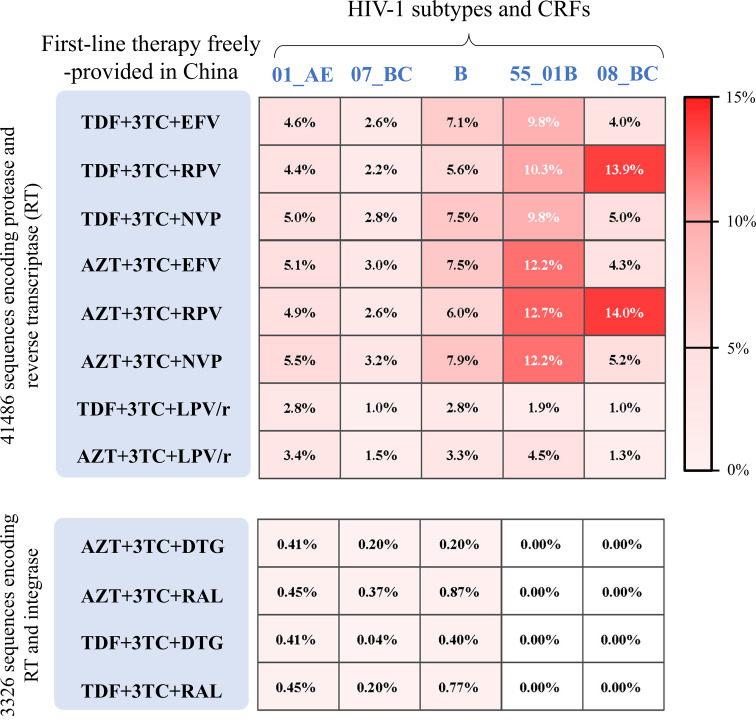
Heat map of average transmitted resistance rates for 12 freely available regimens recommended in China. Average transmitted drug-resistance rates were calculated for 12 antiretroviral regimens using sequence datasets from five major strains (CRF01_AE, CRF07_BC, subtype B, CRF55_01B and CRF08_BC). Eight regimens containing NNRTIs, NRTIs and PIs were evaluated using 41,486 sequences encoding protease and reverse transcriptase. Four regimens containing NNRTIs, NRTIs and INSTIs were analysed using 3,326 sequences encoding reverse transcriptase and integrase. The abbreviations of drug names are provided in Table S4.

We next analysed the resistance rates of each subtype and CRF to 12 freely available antiretroviral regimens. As shown in Fig. 4(a), high resistance of CRF55_01B to six recommended regimens (resistance rate ≥9.8%) was observed. CRF08_BC also conferred a high resistance to TDF+3TC+RPV (resistance rate: 13.9%) and AZT+3TC+RPV (resistance rate: 14.0%). Despite the limited number of integrase-containing sequences (*N*=3,326), low rates (<1%) of resistance were consistently observed over four INSTI-containing regimens (Fig. 4b). Based on sequences available from recent 5 years, the most common resistance mutations included the following: (i) NNRTIs (K103N, V106M and Y181C), (ii) NRTIs (M184V, K65R and M41L), (iii) INSTIs (T97A and E138A) and (iv) PIs (M46L and M46I) (Table S3).

## Discussion

Using a large dataset of HIV-1 sequences, this study presents a comprehensive twenty-year overview (2003–2024) of HIV-1 variant distribution and TDR in China. The key findings are as follows: (i) Long-term surveillance of HIV-1 subtypes and CRFs revealed substantial geographic diversity across provinces, highlighting the need for continuous molecular monitoring. This is particularly important given the limited implementation of HIV-1 genotypic testing in resource-constrained regions due to high costs and insufficient laboratory infrastructure. Optimizing antiretroviral therapy to treat the dominant variants in each province may be especially beneficial in high-burden regions such as Sichuan, Guangxi, Chongqing, Xinjiang, Yunnan and Guizhou. (ii) TDR among treatment-naïve individuals has increased over time, with notable interprovincial differences, especially for NNRTIs. In Yunnan, NNRTI resistance exceeded 10%, indicating the need to revise regional treatment strategies – such as reducing NNRTI use. (iii) Our analysis of resistance to commonly available antiretroviral regimens showed relatively high resistance rates to antiretroviral combinations including AZT+3TC+NVP (6.8%), AZT+3TC+RPV (8.0%), AZT+3TC+EFV (6.4%), TDF+3TC+NVP (6.0%) and TDF+3TC+RPV (7.2%). In contrast, resistance rates were lower for AZT+3TC+DTG (0.22%), AZT+3TC+RAL (0.33%), TDF+3TC+DTG (0.18%) and TDF+3TC+RAL (0.33%). Overall, this study offers an updated and detailed overview of the evolving landscape of HIV-1 variants and TDR in China.

### HIV-1 subtypes and CRFs circulating in China

Our study examined the temporal dynamics of HIV-1 subtypes and CRFs in China over the past two decades. Between 2003 and 2009, subtype B was the dominant strain, but its proportion declined steadily over time. Since 2010, CRF01_AE has overtaken subtype B as the dominant variant. The transmission advantage of CRF01_AE over subtype B might be explained by the association of CRF01_AE with higher viral loads and more rapid disease progression [16]. These findings were consistent with previous studies that reported a shift in HIV-1 variant distribution in China, based largely on city-level data [17,19]. Notably, the spread of CRF07_BC and CRF08_BC was first observed in Zhaotong city, which served as an early transmission hub linking Yunnan province to other regions in China [20]. The increasing prevalence of multiple HIV-1 variants also suggests an ongoing risk that newly emerging CRF strains may become dominant in certain regions [21,22]. In our study, we analysed sequences of the *pol* gene – the gene most routinely sequenced in clinical settings - to identify HIV-1 subtypes and CRFs using two widely used subtyping tools, COMET [23] and REGA [24]. These tools are effective for classifying established HIV-1 subtypes and CRFs such as CRF55_01B when applied to the *pol* sequence datasets [23,25]. Nevertheless, future investigations may consider using full-length HIV-1 genomes for more accurate detection of HIV-1 subtypes and CRFs, because pol-based analyses may miss recombination breakpoints occurring outside the pol region [26,27].

Unlike North America and Western/Central Europe – where subtype B remains dominant [28] – HIV-1 strains circulating in China exhibit high genetic diversity, with extensive co-circulation of recombinant forms [29]. Our study found that more than half of individuals were infected with either CRF01_AE or CRF07_BC from 2020 to 2024 (Fig. 2b). Similar trends of increasing CRF01_AE and other recombinants have been reported in Southeast Asian countries such as Thailand, Cambodia, Vietnam, the Philippines, Malaysia, Singapore and Indonesia [30,31]. After its outbreak in early 2020, the coronavirus disease 2019 (COVID-19) pandemic may have exerted a complex and potentially significant impact on HIV transmission in China [32,33]. We indeed observed distinct patterns in the distribution of dominant HIV-1 subtypes and CRFs before and after 2020 (Fig. 2). A previous study reported that the anti-COVID strategies partially disrupted HIV transmission and slowed its growth in China [32]. Impacts of the COVID-19 pandemic on HIV transmission have been similarly reported in other countries such as the USA [34], Japan [35] and Canada [36].

Our study observed significant geographic heterogeneity of HIV-1 subtypes and CRFs across provinces in China. In Guangdong, most infections involved CRF01_AE, CRF07_BC and CRF55_01B, consistent with earlier findings [37]. A study also reported declining proportions of CRF07_BC, CRF08_BC and CRF55_01B from 2004– 2007 to 2020–2022 [29]. CRF55_01B, initially identified among men who have sex with men (MSM) in Guangdong, has since spread widely [38], likely due to its strong transmission potential [39]. In contrast, subtype B remains the dominant variant in Henan province, followed by CRF01_AE and CRF07_BC, in agreement with literature data [40]. Transmission in Henan has shifted from blood transfusion to sexual contact, with increasing cases linked to homosexual contact [40]. The high prevalence of CRF55_01B and other recombinant forms in Guangdong might be influenced by the role of Guangdong as an economic hub that potentially facilitates both mobility and transmission within the MSM networks [39]. In contrast, Henan displays a mixed epidemic pattern characterizing by persistent subtype B and rising CRF prevalence [40,41]. Given these regional differences, continued variant surveillance is important, especially among high-risk populations.

### TDR in China

Our analysis of 41,486 sequences from treatment-naïve individuals revealed a rising trend in TDR, particularly to NNRTIs. Two NNRTIs – efavirenz and nevirapine – were widely used in first-line antiretroviral regimens in China [42]. A single amino acid substitution (e.g. Y181C) can confer resistance to both efavirenz and nevirapine [43]. We observed the rising NNRTI resistance rates over time, while the resistance to NRTIs and PIs remained relatively stable over the past 5 years, consistent with earlier studies [29]. NNRTI resistance has also been reported as the predominant resistance type in many Chinese cities [44,46]. Notably, the rate of NNRTI resistance in Yunnan exceeded the 10% threshold defined by international guidelines, highlighting the urgent need for drug resistance monitoring and updated treatment strategies. In line with international guidelines, current recommendations in China support replacing efavirenz with dolutegravir in first-line regimens alongside an NRTI backbone [47].

Our study assessed resistance to 12 freely available regimens in China. Resistance rates were low for four NNRTI-containing regimens (AZT+3TC+DTG, AZT+3TC+RAL, TDF+3TC+DTG and TDF+3TC+RAL), supporting their clinical use for treatment-naïve individuals. Moreover, the average resistance score was higher for TDF+3TC+EFV than for TDF+3TC+RPV. This is consistent with the impact of known efavirenz-associated mutations (e.g. K103N/S, V106M, V108I, V179L, G190S/A and P225H), which have less effect on rilpivirine susceptibility [48]. Moreover, different HIV-1 variants exhibited variable resistance patterns to specific combination regimens [6]. For example, the CRF55_01B strain, first identified in 2013 among three epidemiologically unlinked MSM in Guangdong, harbors the V179D/E mutations that confer resistance to certain NNRTI-based regimens [49,50]. Since its discovery, CRF55_01B has become a major circulating HIV-1 strain in China [51]. In addition, the prevalence of transmitted drug resistance (TDR) in the United States is approximately 18.9% [52], further underscoring the need for continuous surveillance in other countries. Taken together, these findings highlight the importance of monitoring HIV-1 diversity and drug-resistant CRFs to improve the clinical management of HIV-1 infection.

### Multiple factors are associated with HIV-1 drug resistance

HIV-1 drug resistance is shaped by multiple factors such as the presence of resistance-associated mutations, high viral levels, low CD4+ T cell counts, advanced clinical stage, treatment regimens and poor adherence [4,53, 54]. First, many amino acid substitutions in HIV-1 genomes are associated with resistance, as supported by our findings and previous publications [48,54]. Second, early initiation of antiviral therapy helps reduce HIV-1 viral loads and slow disease progression [55], but inadequate adherence can result in viral rebound and the selection of resistant strains [56]. Episodic antiretroviral therapy further increases the risk of opportunistic disease or death, mainly due to declining CD4+ T cell counts and rising viral load [57]. Third, a cohort study of 3,640 people with the virologic failure indicated that drug resistance is associated with low baseline CD4+ T cell counts and suboptimal CD4 recovery rate [58]. Therefore, regimens with high genetic barrier to resistance should be prioritized for patients with advanced diseases or poor immune recovery [58].

A high risk of TDR has been recognized for MSM [59,60]. The transmission rate of drug-resistant variants in MSM is higher than that in other groups, driven by factors such as unprotected sex, multiple sex partners and recreational drug use [61]. Future management should target high-risk populations to reduce both new infections and the spread of resistant strains.

### Limitations

This study has several limitations. First, we analysed 81,563 sequences from individuals living with HIV-1. However, these sequences were not randomly sampled. Remote and resource-limited areas may therefore be underrepresented. Future studies should prioritize these under-sampled areas, such as Qinghai, Guizhou and Tibet. Second, while our analyses revealed associations between specific HIV-1 strains and resistance patterns, we did not conduct *in vitro* or *in vivo* experiments to validate mechanistic effects. Nevertheless, previous studies have documented variant-specific resistance to Food and Drug Administration (FDA)-approved drugs [48,62]. Third, our study focused on mutations in the *pol* region; we did not evaluate mutations in other genomic regions that may influence drug resistance [63,64]. Other factors such as low CD4+ T cell counts, advanced clinical stage and poor adherence also exert an impact on drug resistance [4,53, 54]. Addressing these factors will be an important focus for future studies.

## Conclusion

This study characterizes the temporal dynamics of HIV-1 subtype distribution and TDR in China. CRF01_AE and CRF07_BC emerged as the dominant circulating strains after 2020. Notably, we observed a sustained increase in TDR, particularly to NNRTIs. These findings underscore the urgent need to address the growing challenge of TDR. Optimizing first- and second-line antiretroviral regimens – such as incorporating potent integrase inhibitors with high genetic barriers to resistance [5,65] – may help mitigate this issue. Since the adoption of the ‘treat-all’ policy in 2016, China has made significant efforts in providing universal access to effective antiretroviral therapy. However, ongoing surveillance and adaptive treatment strategies remain essential to curb the evolving threat of drug-resistant HIV-1 strains.

## Supplementary material

10.1099/jgv.0.002217 Uncited Supplementary Material 1.

## Abbreviations

ABC: abacavir
AZT: zidovudine
COVID-19: coronavirus disease 2019
CRF: circulating recombinant form
DTG: dolutegravir
EFV: efavirenz
FTC: emtricitabine
HIV: human immunodeficiency virus
HIV-1: human immunodeficiency virus 1
INSTI: integrase strand transfer inhibitor
LPV: lopinavir
LPV/r: lopinavir/ritonavir
MSM: men who have sex with men
NNRTI: non-nucleoside reverse transcriptase inhibitor
NRTI: nucleoside reverse transcriptase inhibitor
NVP: nevirapine
PI: protease inhibitor
RAL: raltegravir
RPV: rilpivirine
3TC: lamivudine
TDF: tenofovir disoproxil fumarate
TDR: transmitted drug resistance

## References

[1] Chen Z, Xu J, Jin X, Wang J, Huang J (2025). Grand challenges on HIV/AIDS in China – the 5th symposium, Yunnan 2024. Emerg Microbes Infect.

[2] Cao W, Hsieh E, Li T (2020). Optimizing treatment for adults with HIV/AIDS in China: successes over two decades and remaining challenges. Curr HIV/AIDS Rep.

[3] Zhao Y, Wei L, Dou Z, Zhao D, Gan X (2023). Changing mortality and patterns of causes of death in HIV-infected patients — China, 2013–2022. China CDC Wkly.

[4] Seyed Alinaghi S, Afsahi AM, Moradi A, Parmoon Z, Habibi P (2023). Current ART, determinants for virologic failure and implications for HIV drug resistance: an umbrella review. AIDS Res Ther.

[5] De Clercq E, Zhang Z, Huang J, Zhang M, Li G (2023). Biktarvy for the treatment of HIV infection: progress and prospects. Biochem Pharmacol.

[6] Li G, Wang Y, De Clercq E (2022). Approved HIV reverse transcriptase inhibitors in the past decade. *Acta Pharmaceutica Sinica B*.

[7] National Center for STD and AIDS Prevention and Control, Chinese Center for Disease Control and Prevention (2023). National Free Antiretroviral Therapy Manual for AIDS, 5th ed.

[8] Zuo L, Liu K, Liu H, Hu Y, Zhang Z (2020). Trend of HIV-1 drug resistance in China: a systematic review and meta-analysis of data accumulated over 17 years (2001–2017). EClinicalMedicine.

[9] Chen H, Hao J, Hu J, Song C, Zhou Y (2023). Pretreatment HIV drug resistance and the molecular transmission network among HIV-positive individuals in China in 2022: multicenter observational study. JMIR Public Health Surveill.

[10] Lessells RJ, Katzenstein DK, de Oliveira T (2012). Are subtype differences important in HIV drug resistance?. Curr Opin Virol.

[11] Nastri BM, Pagliano P, Zannella C, Folliero V, Masullo A (2023). HIV and drug-resistant subtypes. *Microorganisms*.

[12] Rhee S, Kassaye SG, Barrow G, Sundaramurthi JC, Jordan MR (2020). HIV‐1 transmitted drug resistance surveillance: shifting trends in study design and prevalence estimates. J Intern AIDS Soc.

[13] Yue T, Zhang P, Hao Y, He J, Zheng J (2022). Epidemiology and clinical outcomes of HIV infection in south-central China: a retrospective study from 2003 to 2018. Front Public Health.

[14] De Clercq E, Li G, Zhang Y, Huang J, Tan L (2024). Unachieved antiviral strategies with acyclic nucleoside phosphonates: dedicated to the memory of dr. Salvatore “Sam” Joseph Enna. Biochem Pharmacol.

[15] Wang Y, De Clercq E, Li G (2019). Current and emerging non-nucleoside reverse transcriptase inhibitors (NNRTIs) for HIV-1 treatment. Expert Opin Drug Metab Toxicol.

[16] Chu M, Zhang W, Zhang X, Jiang W, Huan X (2017). HIV-1 CRF01_AE strain is associated with faster HIV/AIDS progression in Jiangsu Province, China. Sci Rep.

[17] Xiao P, Li J, Fu G, Zhou Y, Huan X (2017). Geographic distribution and temporal trends of HIV-1 subtypes through heterosexual transmission in China: a systematic review and meta-analysis. Int J Environ Res Public Health.

[18] Lu X, Zhao C, Wang W, Nie C, Zhang Y (2016). HIV-1 genetic diversity and its distribution characteristics among newly diagnosed HIV-1 individuals in Hebei province, China. AIDS Res Ther.

[19] Li X, Li W, Zhong P, Fang K, Zhu K (2016). Nationwide trends in molecular epidemiology of HIV-1 in China. AIDS Res Hum Retroviruses.

[20] Ren L, Wang B, Gong K, Liu P, Zhou S (2017). Epidemiology reveals Zhaotong City as the hub of human immunodeficiency virus type 1 transmission from the Yunnan province to other regions in China. J Gen Virol.

[21] Yu G, Li Y, Huang X, Zhou P, Yan J (2020). Genetic diversity and drug resistance of HIV-1 CRF55_01B in Guangdong, China. Curr HIV Res.

[22] Guan X, Han M, Li Z, Wang L, Zhang D (2020). HIV-1 genetic diversity and transmitted drug resistance among newly diagnosed HIV-1 individuals in Jiangmen, China. J Med Virol.

[23] Struck D, Lawyer G, Ternes AM, Schmit JC, Bercoff DP (2014). COMET: adaptive context-based modeling for ultrafast HIV-1 subtype identification. Nucleic Acids Res.

[24] Pineda-Peña A-C, Faria NR, Imbrechts S, Libin P, Abecasis AB (2013). Automated subtyping of HIV-1 genetic sequences for clinical and surveillance purposes: performance evaluation of the new REGA version 3 and seven other tools. Infect Genet Evol.

[25] Fabeni L, Berno G, Fokam J, Bertoli A, Alteri C (2017). Comparative evaluation of subtyping tools for surveillance of newly emerging HIV-1 strains. J Clin Microbiol.

[26] Coldbeck-Shackley RC, Adamson PJ, Whybrow D, Selway CA, Papanicolas LE (2024). Direct whole-genome sequencing of HIV-1 for clinical drug-resistance analysis and public health surveillance. J Clin Virol.

[27] Li G, Piampongsant S, Faria NR, Voet A, Pineda-Peña A-C (2015). An integrated map of HIV genome-wide variation from a population perspective. Retrovirology.

[28] Williams A, Menon S, Crowe M, Agarwal N, Biccler J (2023). Geographic and population distributions of human immunodeficiency virus (HIV)–1 and HIV-2 circulating subtypes: a systematic literature review and meta-analysis (2010–2021). J Infect.

[29] Liu X, Wang D, Hu J, Song C, Liao L (2023). Changes in HIV-1 subtypes/sub-subtypes and transmitted drug resistance among ART-naïve HIV-infected individuals — China, 2004–2022. China CDC Wkly.

[30] Rafaqat W, Tariq U, Farooqui N, Zaidi M, Raees A (2022). Analysis of temporal changes in HIV-1 CRF01_AE gag genetic variability and CD8 T-cell epitope evolution. PLoS One.

[31] Chen Y, Hora B, DeMarco T, Berba R, Register H (2019). Increased predominance of HIV-1 CRF01_AE and its recombinants in the Philippines. J Gen Virol.

[32] Wang L, Zhao N, Wang Y, Sun K, Wang Y (2023). Impact of the COVID-19 pandemic and the dynamic COVID-zero strategy on HIV incidence and mortality in China. BMC Public Health.

[33] Geng M-J, Zhang H-Y, Yu L-J, Lv C-L, Wang T (2021). Changes in notifiable infectious disease incidence in China during the COVID-19 pandemic. Nat Commun.

[34] Goodreau SM, Delaney KP, Zhu W, Smith DK, Mann LM (2023). Impacts of COVID-19 on sexual behaviors, HIV prevention and care among men who have sex with men: a comparison of New York City and Metropolitan Atlanta. PLoS One.

[35] Nishiura H, Fujiwara S, Imamura A, Shirasaka T (2024). HIV incidence before and during the COVID-19 pandemic in Japan. Math Biosci Eng.

[36] Miller RL, McLaughlin A, Montoya V, Toy J, Stone S (2022). Impact of SARS-CoV-2 lockdown on expansion of HIV transmission clusters among key populations: a retrospective phylogenetic analysis. Lancet Reg Health Am.

[37] Chen Y, Shen Z, Feng Y, Ruan Y, Li J (2021). HIV-1 subtype diversity and transmission strain source among men who have sex with men in Guangxi, China. Sci Rep.

[38] Wang N, Zhong P (2015). Molecular epidemiology of HIV in china: 1985-2015. Zhonghua Liu Xing Bing Xue Za.

[39] Gan M, Zheng S, Hao J, Ruan Y, Liao L (2021). The prevalence of CRF55_01B among HIV-1 strain and its connection with traffic development in China. Emerg Microb Infect.

[40] Yang Z, Wei S, Liu J, Piao J, Xu L (2020). Characterization of HIV-1 subtypes and drug resistance mutations in Henan Province, China (2017–2019). Arch Virol.

[41] Liu J, Fu C, Yang X, Zhang X, Wei S (2025). HIV-1 subtype distribution and drug resistance profiles among PLWHA with detectable viremia in Henan Province, China, 2023. Sci Rep.

[42] Cihlar T, Fordyce M (2016). Current status and prospects of HIV treatment. Curr Opin Virol.

[43] Reuman EC, Rhee SY, Holmes SP, Shafer RW (2010). Constrained patterns of covariation and clustering of HIV-1 non-nucleoside reverse transcriptase inhibitor resistance mutations. J Antimicrob Chemother.

[44] Zhou C, Liang S, Li Y, Zhang Y, Li L (2022). Characterization of HIV-1 molecular epidemiology and transmitted drug-resistance in newly diagnosed HIV-infected patients in Sichuan, China. BMC Infect Dis.

[45] Zeng R, Ren D, Gong X, Wei M, Gao L (2020). HIV-1 genetic diversity and high prevalence of pretreatment drug resistance in Tianjin, China. AIDS Res Hum Retroviruses.

[46] Lan Y, Li L, He X, Hu F, Deng X (2021). Transmitted drug resistance and transmission clusters among HIV-1 treatment-naïve patients in Guangdong, China: a cross-sectional study. *Virol J*.

[47] Gandhi RT, Bedimo R, Hoy JF, Landovitz RJ, Smith DM (2023). Antiretroviral drugs for treatment and prevention of HIV infection in adults: 2022 recommendations of the International Antiviral Society–USA Panel. JAMA.

[48] Wensing AM, Calvez V, Ceccherini-Silberstein F, Charpentier C, Günthard HF (2025). 2025 update of the drug resistance mutations in HIV-1. *Top Antivir Med*.

[49] Han X, An M, Zhang W, Cai W, Chen X (2013). Genome sequences of a novel HIV-1 circulating recombinant form, CRF55_01B, identified in China. Genome Announc.

[50] Gatanaga H, Ode H, Hachiya A, Hayashida T, Sato H (2010). Combination of V106I and V179D polymorphic mutations in human immunodeficiency virus type 1 reverse transcriptase confers resistance to efavirenz and nevirapine but not etravirine. Antimicrob Agents Chemother.

[51] Zhao J, Cai W, Zheng C, Yang Z, Xin R (2014). Origin and outbreak of HIV-1 CRF55_01B among MSM in Shenzhen, China. J Acquir Immune Defic Syndr.

[52] McClung RP, Oster AM, Ocfemia MCB, Saduvala N, Heneine W (2022). Transmitted drug resistance among human immunodeficiency virus (HIV)-1 diagnoses in the United States, 2014–2018. Clin Infect Dis.

[53] Carr A, Mackie NE, Paredes R, Ruxrungtham K (2023). HIV drug resistance in the era of contemporary antiretroviral therapy: a clinical perspective. Antivir Ther.

[54] Pang X, He Q, Tang K, Huang J, Fang N (2024). Drug resistance and influencing factors in HIV-1-infected individuals under antiretroviral therapy in Guangxi, China. J Antimicrob Chemother.

[55] Kumar S, Batra H, Singh S, Chawla H, Singh R (2020). Effect of combination antiretroviral therapy on human immunodeficiency virus 1 specific antibody responses in subtype-C infected children. J Gen Virol.

[56] Shubber Z, Mills EJ, Nachega JB, Vreeman R, Freitas M (2016). Patient-reported barriers to adherence to antiretroviral therapy: a systematic review and meta-analysis. PLoS Med.

[57] El-Sadr WM, Lundgren JD, Neaton JD, Gordin F, Abrams D (2006). CD4+ count-guided interruption of antiretroviral treatment. N Engl J Med.

[58] Stirrup OT, Sabin CA, Phillips AN, Williams I, Churchill D (2019). Associations between baseline characteristics, CD4 cell count response and virological failure on first-line efavirenz + tenofovir + emtricitabine for HIV. J Virus Erad.

[59] Kassaye SG, Grossman Z, Balamane M, Johnston-White B, Liu C (2016). Transmitted HIV drug resistance is high and longstanding in metropolitan Washington, DC. Clin Infect Dis.

[60] Trebelcock WL, Lama JR, Duerr A, Sanchez H, Cabello R (2019). HIV pretreatment drug resistance among cisgender MSM and transgender women from Lima, Peru. J Intern AIDS Soc.

[61] Zhang J, Guo Z, Pan X, Zhang W, Yang J (2017). Highlighting the crucial role of Hangzhou in HIV-1 transmission among men who have sex with men in Zhejiang, China. Sci Rep.

[62] Santoro MM, Perno CF (2013). HIV-1 genetic variability and clinical implications. ISRN Microbiol.

[63] Li G, Theys K, Verheyen J, Pineda-Peña A-C, Khouri R (2015). A new ensemble coevolution system for detecting HIV-1 protein coevolution. Biol Direct.

[64] Nouhin J, Tzou PL, Rhee S-Y, Sahoo MK, Pinsky BA (2023). Human immunodeficiency virus 1 5’-leader mutations in plasma viruses before and after the development of reverse transcriptase inhibitor-resistance mutations. J Gen Virol.

[65] Zhao AV, Crutchley RD, Guduru RC, Ton K, Lam T (2022). A clinical review of HIV integrase strand transfer inhibitors (INSTIs) for the prevention and treatment of HIV-1 infection. *Retrovirology*.

[66] Hao J, Liu X, Wang D, Hu H, Li F (2025). Transmitted HIV-1 drug resistance among newly diagnosed individuals in 31 provincial-level administrative divisions in China in 2023: a cross-sectional survey. Clin Infect Dis.

