# Supplementary material for: Geographical and temporal trends of HIV-1 subtypes and drug resistance in China: a nationwide study over two decades (2003–2024)
Source: J Gen Virol. 2026 Feb 2;107(2):002217. doi: 10.1099/jgv.0.002217 (PMC12865022; doi:10.1099/jgv.0.002217)
Supplement: Uncited Supplementary Material 1. [file jgv-107-02217-s001.pdf]

Table S1: Literature results of dominant HIV-1 subtypes or CRFs in Chinese provinces

Province name	Year	Dominant subtype/CRF	Proportion	References
Gansu	2020-2021	CRF07_BC	176 (58.1%)	Qiao RJ, Zhang LC, Feng Y, Tu AX, Wang XR, Shi L, et al. [Characteristics of the pretreatment drug resistance and molecular transmission network in newly reported HIV infections in some areas of Gansu Province from 2020 to 2021]. Chin J AIDS STD. 2023;29(8):860-865. DOI:10.13419/j.cnki.aids.2023.08.04
		CRF01_AE	64 (21.1%)	
		B	21 (6.9%)	
		CRF55_01B	16 (5.3%)	
		Others	26 (8.6%)	
Guizhou	2007	CRF01_AE	62 (52.1%)	Sun XG, He X, Li ZJ, Zhang XH, Shen LM. [The Study of characteristic of new HIV-1 subtypes in Guizhou]. Chongqing Medicine. 2011;40(29):2922-2924.
		CRF07_BC	49 (41.2%)	
		B	8 (5.0%)	
		CRF08_BC	2 (1.7%)	
Ningxia	2012-2020	CRF07_BC	239 (60.05%)	Song L, Yang DZ, Cao M, Zhao LH, Li HJ. [Analysis of molecular transmission network characteristics of HIV-1 infected person in Ningxia from 2012 to 2020]. Ningxia Med J. 2022;44(06):504-508. DOI:10.13621/j.1001-5949.2022.06.0504.
		CRF01_AE	103 (25.88%)	
		B	30 (7.54%)	
		CRF08_BC	11 (2.76%)	
		CRF55_01B	11 (2.76%)	
		C	4 (1.01%)	
Jilin	2022	CRF01_AE	125 (60.68%)	Guo Qi WA, Feng Yi,Wu Donglin,Qi Xiaochen,Wang Hui,Zang Xihui,Liu Sihan, Xing Hui, Sun Liuyan: Characteristics of drug-resistance and molecular transmission network among pre-treatment HIV1 infected patients in Jilin province. *International Journal of Virology* 2024, 31:270-274
		CRF07_BC	62 (30.10%)	
		B	11 (5.34%)	
		CRF55_01B	4 (1.94%)	
		CRF02_AG	3 (1.46%)	
		CRF08_BC	1 (0.49%)	
Hunan	2023	CRF07_BC	202 (41.22%)	HU Min,ZOU Xiao-bai,HE Jian-mei,ZHENG Jun,TANG Qi,CHEN Xi. Study on the distribution characteristics of HIV strain subtypes among newly
		CRF01_AE	156(31.84%)	
		CRF08_BC	44 (8.98%)	

		CRF55_01B	21 (4.29%)	reported infections in Hunan Province in 2023[J],2025,52(01):160-166.DOI:10.20043/j.cnki.MPM.202407257
		B	21 (4.29%)	
		CRF85_BC	12 (2.45%)	
		C	9(1.84%)	
Taiwan	2000 -2016	B	858 (53.5%)	Jen IA, Li WY, Wu SJ, Chen PMT, Liu WL, Yen YF, Fann CSJ, Chen YA: Molecular epidemiology and long-term survival analysis of HIV-1/AIDS patients infected with CRF07_BC, CRF01_AE and subtype B in Taiwan. *PLoS One* 2025, 20(6):e0323250.
		CRF07_BC	690 (43.0%)	
		CRF01_AE	57 (3.5%)	
Shanghai	2021	CRF07_BC	52 (36.36%)	Jia M, Li JJ, Hong L,Tao J. [Analysis on HIV-1 genotype distribution and drug resistance in Hongkou district, Shanghai, 2021]. DISEASE SURVEILLANCE. 2024;39(07):869-874.
		CRF01_AE	49 (34.27%)	
		C	13 (9.09%)	
		B	9 (6.29%)	
		CRF55_01B	4 (2.80%)	
Fujian	2020	CRF07_BC	488 (52.1%)	Xie MR, Lin LY, Wang ZH, Qiu YF, Lu XL, Zhang CH, et al. [Molecular epidemiological characteristics of newly diagnosed HIV-1 cases in Fujian Province in 2020]. Chin J Schisto Control. 2023;35(06):583-589.DOI:10.16250/j.32.1374.2023003.
		CRF01_AE	285 (30.4%)	
		CRF08_BC	46 (2.1%)	
		CRF55_01B	28 (1.7%)	
Inner Mongol	2021	CRF07_BC	85 (46.70%)	Ran KF. 2022. Study on Molecular Epidemiology and Pretreatment Drug Resistance of HIV-1 in Inner Mongolia in 2021 [Master's thesis]. Hohhot: Inner Mongolia Medical University.
		CRF01_AE	76 (41.76%)	
		CRF55_01B	5 (2.75%)	
		Others	16 (8.79%)	
	2015	CRF01_AE	-	Wang Y, Yang JH, Yang H, Yang JY, Gao Y, Liu J. [Analysis of drug-resistant of HIV-1 strains among antiretroviral-therapy failure patients in Inner Mongolia]. Chin J Health Lab Tee. 2021;31(19):2352-2356.
Tibet	2003-2018	CRF 07_BC	-	Zhao J, Lv X, Chang L, Ji H, Harris BJ, Zhang L, et al. HIV-1 molecular epidemiology and drug resistance-associated mutations among treatment-

				naïve blood donors in China. Sci Rep. 2020;10(1):7571. DOI:10.1038/s41598-020-64463-w.
	2019-2023	CRF 07_BC	-	Zhao J, Lv X, Chang L, Ji H, Harris BJ, Zhang L, et al. HIV-1 molecular epidemiology and drug resistance-associated mutations among treatment-naïve blood donors in China. Sci Rep. 2020;10(1):7571. DOI:10.1038/s41598-020-64463-w.<br>Ye J, Chen J, Wang J, Wang Y, Xing H, Yu F, et al. CRF07_BC is associated with slow HIV disease progression in Chinese patients. Sci Rep. 2022;12(1):3773. DOI:10.1038/s41598-022-07518-4.<br>Wang D, Feng Y, Hao J, Hu H, Li F, Li J, et al. National and Regional Molecular Epidemiology of HIV-1 - China, 2004-2023. China CDC Wkly. 2024;6(48):1257-1263. DOI:10.46234/ccdcw2024.252.
Qinghai	2003-2018	CRF 07_BC	-	Zhao J, Lv X, Chang L, Ji H, Harris BJ, Zhang L, et al. HIV-1 molecular epidemiology and drug resistance-associated mutations among treatment-naïve blood donors in China. Sci Rep. 2020;10(1):7571. DOI:10.1038/s41598-020-64463-w.
Qinghai	2019-2023	CRF 01_AE	-	Ye J, Chen J, Wang J, Wang Y, Xing H, Yu F, et al. CRF07_BC is associated with slow HIV disease progression in Chinese patients. Sci Rep. 2022;12(1):3773. DOI:10.1038/s41598-022-07518-4.
Jiangxi	1994-2020	CRF 01_AE	-	Ye J, Chen J, Wang J, Wang Y, Xing H, Yu F, et al. CRF07_BC is associated with slow HIV disease progression in Chinese patients. Sci Rep. 2022;12(1):3773. DOI:10.1038/s41598-022-07518-4.
Shandong	2007-2016	CRF 07_BC	-	Yang GG, Zhang N, Hao LZ, Huang PX, Yu YH, Hu J, et al. [Analysis on the epidemic features of male HIV-infected and AIDS patients by sexual transmission in Shandong Province from 2007 to 2016]. Chinese Journal of Preventive

				Medicine. 2018;52(3):292-295. DOI:10.3760/cma.j.issn.0253-9624.2018.03.014
	2019-2023	CRF 07_BC	-	Zhang R, Dong TL, Liang WL, Cao ZB, Xie Z, Liu KM, at al. [Analysis of HIV-1 genetic subtype and pretreatment drug resistance among men who have sex with men infected with HIV-1 from 19 cities of 6 provinces in China]. Chinese Journal of Epidemiology. 2022;43(04):523-527. DOI:10.3760/cma.j.cn112338-20211125-00918
Xinjiang	2020-2023	CRF07_BC	-	Hu HP, Hao JJ, Liu X, Chen HL, Hu J, Song C, et al. [Prevalence of HIV-1 integrase inhibitor resistance before antiviral therapy in some provinces of China in 2022]. International Journal of Virology. 2024;31(4):265-269. DOI:10.3760/cma.j.issn.1673-4092.2024.04.001. Huang YD, Wang FY, Wang JL, Jin T, Zhao YY, Li H, et al. [Drug Resistance and Molecular Network Analysis of HIV/AIDS Patients with Antiviral Therapy Failure in Aksu Prefecture, Xinjiang Uygur Autonomous Region].Chinese Journal of Virology. 2024;40(04):862-868.DOI:10.13242/j.cnki.bingduxuebao.004550

Table S2: Temporal distribution of resistance sequences for five drug classes

Years	NNRTIs	NRTIs	PIs	INSTIs	Total
	N	N	N	N	N
2003	7	3	1	2	126
2004	8	5	2	0	150
2005	5	0	1	0	179
2006	17	6	0	1	177
2007	31	6	0	0	610
2008	13	6	1	0	345
2009	97	54	6	6	1660
2010	62	22	1	0	1774
2011	69	20	7	0	1942
2012	63	30	3	0	2670
2013	114	47	8	0	4176
2014	95	31	7	0	2771
2015	154	46	12	0	4264
2016	72	35	7	0	2108
2017	337	181	9	1	4561
2018	323	158	13	0	3721
2019	335	144	8	3	3805
2020	186	202	2	6	1824
2021	223	66	1	9	2265
2022	132	24	3	1	1127
2023*	1	0	0	0	7
2024	87	24	1	0	1224

*: Because fewer than 10 sequences were available in 2023 in our dataset, resistance data from the publication [1] were used in our manuscript to supplement that year.

Table S3: Resistance-associated mutations in our dataset of *pol*-containing sequences in China from 2020-2024

Mutation sites	Frequency (N)	Rate (%)
**NNRTIs**		
L100I	3	<0.1%
K101E	28	0.43%
K101P	6	<0.1%
K103N	199	3.09%
V106M	55	0.85%
V108I	17	0.26%
E138A	37	0.57%
E138Q	28	0.43%
E138G	5	<0.1%
Y181C	44	0.68%
Y188L	17	0.26%
Y188H	4	<0.1%
G190A	26	0.40%
G190S	24	0.37%
P225H	21	0.33%
F227L	19	0.30%
M230L	9	0.14%
L100I	3	<0.1%
**NRTIs**		
M41L	23	0.36%
K65R	57	0.88%
D67N	22	0.34%
D67G	4	<0.1%
K70E	14	0.22%
K70R	5	<0.1%
L74I	6	<0.1%
L74V	5	<0.1%
Y115F	19	0.30%
M184V	95	1.47%
M184I	15	0.23%
L210W	10	0.16%
T215F	3	<0.1%
T215Y	7	0.11%
T215S	7	0.11%
K219E	8	0.12%
K219Q	8	0.12%
**Protease inhibitors**		
L23I	2	<0.1%
V32I	1	<0.1%

M46I	17	0.26%
M46L	16	0.25%
F53L	3	<0.1%
I54L	1	<0.1%
G73S	2	<0.1%
V82A	3	<0.1%
N83D	4	<0.1%
I84V	1	<0.1%
I85V	8	0.12%
N88D	2	<0.1%
L90M	3	<0.1%
**INSTIs**		
E157Q	17	0.26%
L74M	8	0.12%
T97A	4	<0.1%
E138A	2	<0.1%
E138K	2	<0.1%
Q148R	2	<0.1%
S147G	2	<0.1%
T66I	2	<0.1%
G140A	1	<0.1%
G140S	1	<0.1%
G118R	1	<0.1%
E92Q	1	<0.1%
R263K	1	<0.1%

Table S4: Abbreviations of anti-HIV drugs in our study

Drug name	Abbreviations	Categories
Tenofovir disoproxil fumarate	TDF	NRTI
Lamividine	3TC	NRTI
Emtricitabine	FTC	NRTI
Efavirenz	EFV	NNRTI
Abacavir	ABC	NRTI
Zidovudine	AZT	NRTI
Nevirapine	NVP	NNRTI
Rilpivirine	RPV	NNRTI
Lopinavir /ritonavir	LPV/r	PI
Dolutegravir	DTG	INSTI
Raltegravir	RAL	INSTI

NRTI, nucleoside reverse transcriptase inhibitor;

NNRTI, non-nucleoside reverse transcriptase inhibitor

INSTI, integrase strand transfer inhibitor

PI, protease inhibitor

References

1. Hao J, Liu X, Wang D, Hu H, Li F, Li Y, Hu J, Song C, Ruan Y, Feng Y *et al*: **Transmitted HIV-1 Drug Resistance Among Newly Diagnosed Individuals in 31 Provincial-Level Administrative Divisions in China in 2023: A Cross-sectional Survey**. *Clin Infect Dis* 2025, **81**(3):531-538.
